# Hyper-Expression of PD-1 Is Associated with the Levels of Exhausted and Dysfunctional Phenotypes of Circulating CD161^++^TCR iVα7.2^+^ Mucosal-Associated Invariant T Cells in Chronic Hepatitis B Virus Infection

**DOI:** 10.3389/fimmu.2018.00472

**Published:** 2018-03-19

**Authors:** Yean K. Yong, Alireza Saeidi, Hong Y. Tan, Mohamed Rosmawati, Philip F. Enström, Rami Al Batran, V. Vasuki, Indranil Chattopadhyay, Amudhan Murugesan, Ramachandran Vignesh, Adeeba Kamarulzaman, Jayakumar Rajarajeswaran, Abdul W. Ansari, Jamuna Vadivelu, James E. Ussher, Vijayakumar Velu, Marie Larsson, Esaki M. Shankar

**Affiliations:** ^1^Laboratory Center, Xiamen University Malaysia, Sepang, Malaysia; ^2^Department of Medicine, University of Malaya Medical Centre, Kuala Lumpur, Malaysia; ^3^China-ASEAN Institute of Marine Science (CAMS), Xiamen University Malaysia, Sepang, Malaysia; ^4^Department of Traditional Chinese Medicine, Xiamen University Malaysia, Sepang, Malaysia; ^5^Division of Molecular Virology, Department of Clinical and Experimental Medicine, Linköping University, Linköping, Sweden; ^6^Department of Medical Microbiology, Faculty of Medicine, University of Malaya, Kuala Lumpur, Malaysia; ^7^Department of Microbiology, The Government Thiruvarur Medical College and Hospital, Thiruvarur, India; ^8^Division of Molecular Cancer Biology, Department of Life Sciences, Central University of Tamil Nadu, Thiruvarur, India; ^9^The Government Theni Medical College and Hospital, Theni, India; ^10^Laboratory-Based Department, Universiti Kuala Lumpur, Ipoh, Malaysia; ^11^Center of Excellence for Research in AIDS, University of Malaya, Kuala Lumpur, Malaysia; ^12^Department of Molecular Medicine, Faculty of Medicine, University of Malaya, Kuala Lumpur, Malaysia; ^13^Department of Microbiology and Immunology, University of Otago, Dunedin, New Zealand; ^14^Department of Microbiology and Immunology, Emory Vaccine Center, Atlanta, GA, United States; ^15^Division of Infection Biology, Department of Life Sciences, Central University of Tamil Nadu, Thiruvarur, India; ^16^Department of Microbiology, Central University of Tamil Nadu, Thiruvarur, India

**Keywords:** HBV infection, HLA-DR, immune exhaustion, immunosenescence, mucosal-associated invariant T cells, PD-1, CTLA-4

## Abstract

Mucosal-associated invariant T (MAIT) cells, defined as CD161^++^TCR iVα7.2^+^ T cells, play an important role in the innate defense against bacterial infections, and their functionality is impaired in chronic viral infections. Here, we investigated the frequency and functional role of MAIT cells in chronic hepatitis B virus (HBV) infection. The peripheral CD3^+^CD161^++^TCR iVα7.2^+^ MAIT cells in chronic HBV-infected patients and healthy controls were phenotypically characterized based on CD57, PD-1, TIM-3, and CTLA-4, as well as HLA-DR and CD38 expression. The frequency of MAIT cells was significantly decreased among chronic HBV-infected individuals as compared to controls. Expression of CD57, PD-1, CTLA-4, as well as HLA-DR and CD38 on MAIT cells was significantly elevated in chronic HBV-infected individuals relative to controls. The percentage of T cell receptor (TCR) iVα7.2^+^ CD161^+^ MAIT cells did not correlate with HBV viral load but inversely with HLA-DR on CD4^+^ T cells and MAIT cells and with CD57 on CD8^+^ T cells suggesting that decrease of MAIT cells may not be attributed to direct infection by HBV but driven by HBV-induced chronic immune activation. The percentage and expression levels of PD-1 as well as CTLA-4 on MAIT cells inversely correlated with plasma HBV-DNA levels, which may suggest either a role for MAIT cells in the control of HBV infection or the effect of HBV replication in the liver on MAIT cell phenotype. We report that decrease of TCR iVα7.2^+^ MAIT cells in the peripheral blood and their functions were seemingly impaired in chronic HBV-infected patients likely because of the increased expression of PD-1.

## Introduction

Hepatitis B virus (HBV) infection remains a major threat worldwide with ~240 million people chronically infected, and estimates suggest that ~780,000 die annually due to complications including cirrhosis, hepatocellular carcinoma (HCC), and chronic liver failure (1). Therefore, reducing the pool of HBV-infected individuals is of paramount importance for public health. Despite the availability of an effective prophylactic vaccine, a smaller fraction of vaccinated individuals (~2%) in endemic regions can still be chronically infected with HBV (2). Furthermore, prophylactic vaccines have little or no effect on those who have already been chronically infected with HBV (3, 4).

The therapeutic goal in chronic HBV infection is to achieve sustained suppression of viral replication and thereby reduce liver disease progression and the risk of clinical end-points. Despite potent antiviral drugs that inhibit HBV reverse transcriptase, “complete” virological response, defined as hepatitis B surface antigen (HBsAg) clearance, is rarely achieved (5–7). The HBV genome is present in the hepatocytes in a mono-chromosomal form, i.e., cccDNA, and remains intact in spite of the use of these anti-HBV drugs (8). Therefore, virus eradication is seldom achieved and patients experience viral rebound after withdrawal of antiviral therapy (2).

In the absence of treatment, failure to achieve sustained viral control is associated with sub-optimal antiviral CD4^+^ and CD8^+^ T-cell responses against HBV core, polymerase protein (9, 10), and antibody responses to envelope (11). Consequently, failure to elicit effective immune responses may be due to clonal deletion of HBV-specific T cell responses (12) and functional exhaustion resulting from the upregulation of co-inhibitory molecules, for instance PD-1 (13), CTLA-4 (14), and TIM-3 (15, 16). Hence, there is a pressing need to better understand the virus–host interactions and the immunological mechanisms in chronic HBV infection, which allows for the designing of novel immunotherapeutic approaches to boost antiviral immune responses.

Mucosal-associated invariant T (MAIT) cells are a recently described subset of innate-like T cells that comprise ~5% of the T-cell pool of adult peripheral blood and are enriched to ~40% in mucosal tissues, liver, and lung in healthy individual (17–19). MAIT cells express a semi-invariant T cell receptor (TCR), including Vα7.2 coupled with restricted Jα segments (Jα33, Jα12, or Jα20), and limited Vβ repertoires (20, 21) as well as their high expression of CD161 (17, 22). MAIT cells play an important role in innate host defense against bacterial infections and are either depleted and/or exhausted in chronic viral infections, including HIV (23–25), chronic HBV (26), and HCV infection (22, 27, 28), and TB infection (29, 30). Here, we investigated the frequency and functional role of MAIT cells along with those of CD4^+^ and CD8^+^ T cells in chronic HBV infection. We characterized the phenotypes of peripheral CD3^+^CD161^++^TCR iVα7.2^+^ MAIT cells in chronic HBV-infected patients with and without HBV-DNAemia as compared to healthy controls (HCs) based on the expression of co-inhibitory and senescence markers, CD57, PD-1, TIM-3, and CTLA-4, as well as, HLA-DR and CD38.

## Materials and Methods

### Ethics Statement

The protocols involving human subjects were approved by the Medical Ethics Committee (MEC) of Universiti Malaya Medical Centre (UMMC), Kuala Lumpur, Malaysia (MEC201311-0496), and conducted as per the guidelines of the International Conference on Harmonization Guidelines and Declaration of Helsinki. All human subjects were adults and provided written informed consent. The written consent form was approved by the ethics committee and signed by the subject or the subject’s legally authorized representative. A copy of the document was given to the person signing the form. The entire consent process was approved by the MEC for conduct of the research.

### Study Subjects

A total of 21 individuals with chronic HBV (CHB) infection with HBV-DNAemia positive (limit of detection, 19 IU equivalent to 120 copies/ml) (CHB DNA +ve, *n* = 12) and HBV-DNAemia negative (CHB DNA −ve, *n* = 9) as well as HCs (*n* = 13) were recruited for a cross-sectional analysis. Peripheral blood was obtained from HBV-infected subjects at the Hepatology Unit of the UMMC, Malaysia. HCs were defined as individuals free from chronic viral (HBV, HCV, and HIV) and *Mycobacterium tuberculosis* infections. HBV plasma viral loads were measured using a commercial COBAS AmpliPrep-COBAS TaqMan HBV test (CAP-CTM; Roche Molecular Systems, Inc., Branchburg, NJ, USA). Plasma alanine transaminase (ALT) level in the patients was measured on a Hitachi7050 Automatic Analyzer (Hitachi Corp., Tokyo, Japan) using a commercial ALT assay kit (Wako Pure Chemicals, Osaka, Japan). The cutoff values were set at 20 ng/ml.

### Peripheral Blood Mononuclear Cells (PBMCs)

Ten millilitres of venous blood was collected by venipuncture in lithium heparin BD Vacutainer (BD Biosciences, Franklin Lakes, NJ, USA) tubes and was kept at room temperature. PBMCs were extracted by density-gradient centrifugation using Ficoll Paque Plus (Sigma-Aldrich, St. Louis, MO, USA) overlay within 4 h post-collection. Cell viability was assessed by 0.4% trypan blue vital staining. Purified PBMCs were subsequently used in the immunophenotyping and cell culture experiments.

### MAIT Cell Activation and Intracellular Staining

For intracellular cytokine staining, the cells were stimulated with PMA (100 ng/ml) and ionomycin (0.67 μM) for 5 h at 37°C and 5% CO_2_ prior to immunostaining. Brefeldin A (10 g/ml) was added for the last 4 h of stimulation. The immunostained samples were washed twice prior to acquisition on a FACS Canto II Immunocytometry system (BD Biosciences).

### Multicolor Flow Cytometry

All antibodies were pre-titrated to determine appropriate working concentrations. All antibodies were purchased from BD Pharmingen™ (BD Biosciences) unless otherwise specified. Immunostaining was performed with two panels for surface markers, where the first one included fluorescein isothiocyanate (FITC)-conjugated anti-CD57, phycoerythrin (PE)-conjugated anti-TCR-Va7.2 (MiltenyiBiotec), peridinin chlorophyll protein (PerCp)-Cy5.5-conjugated anti-CD3, PE-Cy7-conjugated anti-TIM3 (eBioscience), allophycocynanin (APC)-conjugated anti-CD161, APC-H7-conjugated anti-CD8, V500-conjugated CD4, and brilliant violet 421 (BV421)-conjugated anti-CD279 (PD-1). The second panel was performed with FITC-conjugated anti-HLA-DR, PE-conjugated anti-CD38, PerCP-Cy5.5-conjugated anti-CD3, PE-conjugated anti-TCR-Va7.2-Vio770 (MiltenyiBiotec), APC-conjugated anti-CD161, APC-H7-conjugated anti-CD8, V500-conjugated CD4, and BV-421-conjugated anti-CTLA-4. The functional assays were stained using two panels; one with FITC-conjugated anti-IFN-γ, PerCp-Cy5.5-conjugated anti-CD3, APC-conjugated anti-CD161, APC-H7-conjugated anti-CD8, PE-conjugated anti-perforin (eBioscience), BV421-conjugated anti-Granzyme-B, and the other with FITC-conjugated anti-IFN-γ, PE, PerCp-Cy5.5-conjugated anti-CD3, APC-conjugated anti-CD161, APC-H7-conjugated anti-CD8, PE-conjugated anti-TNF-alpha (R&D), and PE-Vio770-conjugated anti-TCRVα7.2 (MiltenyiBiotec). Unstained PBMCs and control samples incubated with isotype-matched antibodies of irrelevant specificity were used as controls. After adding the antibodies, the cells were incubated at 4°C in the dark for 30 min and washed twice with washing buffer at 4°C. Finally, 350 μl of washing buffer (PBS, 1% BSA or 10% FBS, 0.1% sodium azide) was added to each tube. The sample tubes were analyzed using a BD FACS Canto II flow cytometer within 1 h post-staining. Flow cytometry analysis was made using FlowJo for Windows, version 10.0.8 (FlowJo LLC, Ashland, OR, USA).

### Statistical Analysis

The primary analysis was to compare the percentages and expression levels (mean fluorescence intensity, MFI) of biomarkers on different subsets of T cells and MAIT cells, and compare between the three study groups. Difference between categorical variables were tested using chi-square test or Fisher’s exact test, whereas continuous variables were tested using the non-parametric Kruskal–Wallis test for multiple group comparisons. If *P*-values were <0.05, 3-way comparisons were subsequently performed separately using Mann–Whitney *U* tests between the three patient groups applying the Benjamini–Hochberg correction for multiple comparisons. Correlation between two continuous variables was compared using the Spearman’s rank correlation. Differences were considered significant with *P < 0.05, ** < 0.01, *** < 0.001, and **** < 0.0001. All analyses and graphs were performed using GraphPad Prism 6 software (GraphPad, La Jolla, CA, USA).

## Results

### Patient Cohort Characteristics

The three groups, non-randomized study design consisted of samples from 21 subjects with chronic HBV infection: Group 1 (G1) 12 CHB DNAemia positive, G2 with 9 CHB DNAemia negative, and a control group (G3) of 13 HCs. The specimens were collected between April and August 2015. CHB infection was defined as subjects who tested positive for HBsAg for at least 6 months after the onset of acute infection (1). There were no significant differences for demographic, laboratory, and clinical data among the three study groups (Table 1).

**Table 1:** Demography, laboratory, and clinical characteristics of study participants.

	HBV-DNA +ve (*n* = 12)	HBV-DNA −ve (*n* = 9)	HC (*n* = 13)	*P* value
Age, year	50 (47.3–57.3)	55 (38–61)	44 (40–51)	0.094
Gender, male (%)	50	66.7	61.5	0.721
Plasma HBV-DNA (copies/mL)	20, 377 (3,468–326,353)	–	–	–
Alanine transaminase (U/L)	26.5 (18.3–40.3)	45 (20–36.5)	–	0.776
Albumin (g/dL)	42.5 (39.3–45)	45 (41–48.5)	–	0.766
HBeAg positive (%)	16.7	11.1	–	0.751
Hepatitis B surface antigen positive (%)	100	100	–	1

*All data are expressed as median (IQR) unless specified. *P-values* are calculated by Fisher exact test for categorical variable and Kruskal–Wallis test for continuous variables. IQR, interquartile range; HBV-DNA +ve, referring to group of patients who chronically infected by hepatitis B virus (HBV) with HBV-DNAemia; HBV-DNA −ve, referring to group of patients who chronically infected by HBV without HBV-DNAemia; and HC, healthy controls*.

### Chronic HBV Infection Is Associated with Increased Immunosenescence and Exhaustion in CD4^+^ and CD8^+^ T Cells

**Figure 1:**
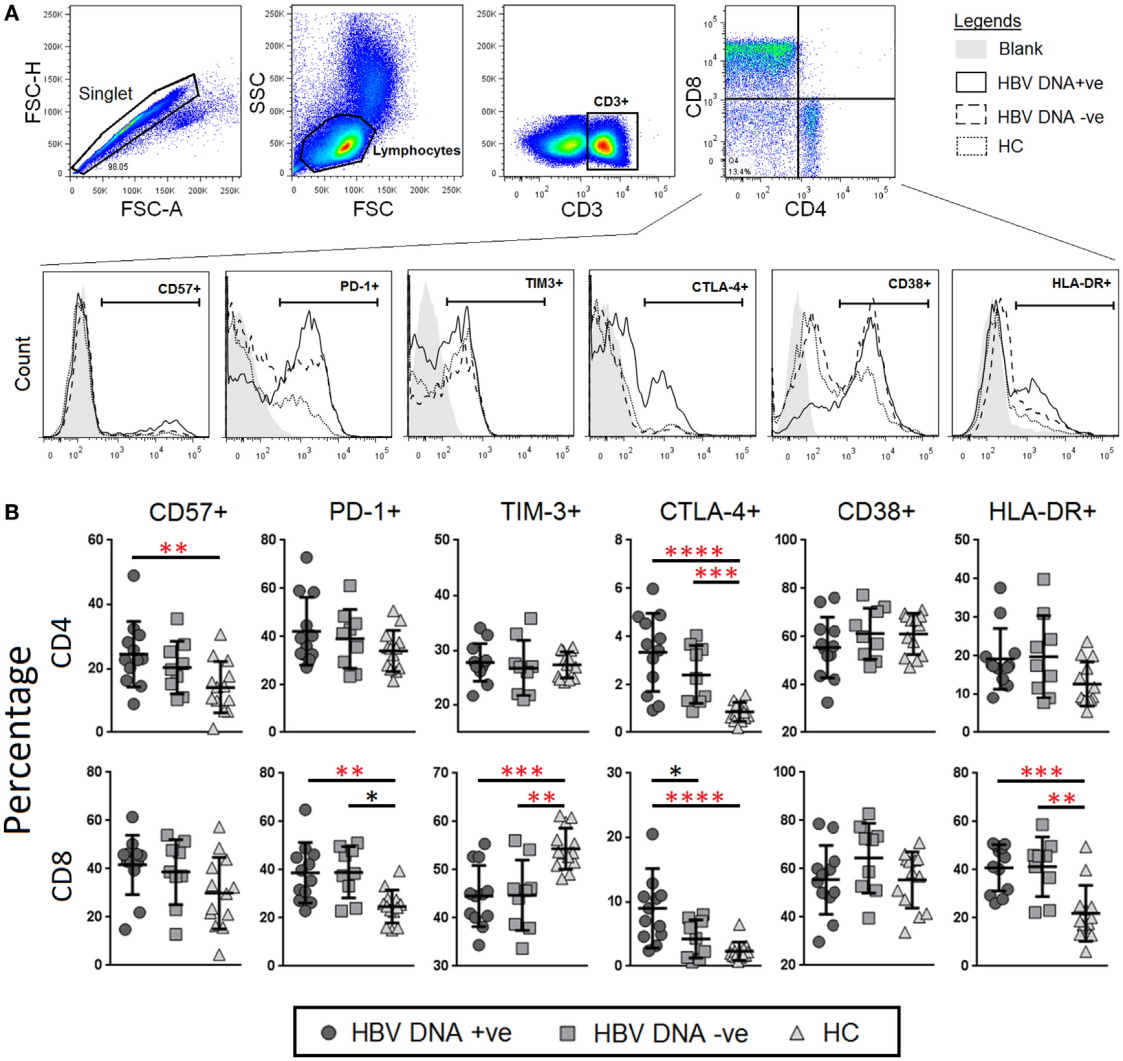
Frequencies and levels of immune senescence, exhaustion, and activation markers expressed on CD4^+^ and CD8^+^ T-cells between chronic hepatitis B virus (HBV)-infected patients with (circle) and without (square) HBV-DNAemia and healthy control (HC) (triangle). **(A)** The gating strategy to identify **(B)** expression levels of CD57, PD-1, TIM-3, CTLA-4 CD38, and HLA-DR on CD4^+^ and CD8^+^ T-cells. Levels of surface markers were compared across the three patient groups and *post hoc* Mann–Whitney *U* tests were then performed for those biomarkers with a Kruskal–Wallis test *P* value of <0.05 (*P < 0.05, **<0.01, ***<0.001, and ****<0.0001). *P*-values remained significant after Benjamini–Hochberg correction of multiple comparisons (marked in red*).

T-cell co-inhibition plays a critical role in T-cell dysfunction during chronic viral infections (31). In order to characterize T-cell co-inhibition during chronic HBV infection, we measured the percentages and expression levels of markers of immunosenescence, i.e., CD57, immune exhaustion, i.e., PD-1, TIM-3, and CTLA-4, as well as chronic immune activation, i.e., CD38, and HLA-DR, in both CD4^+^ and CD8^+^ T cells, and compared between the three study groups (Figure 1A). We found that there was ~twofold higher frequencies of CD4^+^ T cells that expressed CD57 (median = 27.5%) in the HBV-DNA +ve group as compared to HCs (14%) (Figure 1B). The percentage of PD-1^+^CD8^+^ T cells was higher in both groups of CHB patients (median = 38.5 and 36.5%, respectively) as compared to HCs (median = 25%). This result was consistent with the expression levels of PD-1 (measured as MFI) where the MFI of PD-1 were also significantly higher in CD8^+^ T cells in both CHB groups (1.4- and 1.27-fold higher in CHB DNA +ve and DNA −ve, respectively) as compared to HCs. The expression of PD-1 on CD4^+^ T cells was not significantly different among the three study groups. In addition, the percentages and expression of CTLA-4 was higher on both CD4^+^ and CD8^+^ T cells of CHB patients compared to HCs. The percentage and expression of CD38 was lower in CD8^+^ T cells of CHB patients relative to HCs; while the percentages of HLA-DR were significantly higher in CHB patients as compared to HCs. Collectively, our data suggest that there is an increase in immune senescence and exhaustion across both CD4^+^ and CD8^+^ T cells at varying levels in CHB patients (Figure 1B).

### Chronic HBV Infection Was Associated with Depletion of TCR iVα7.2^+^ MAIT Cells Together with Increased Frequencies and Expression of PD-1 and CTLA-4 on TCR iVα7.2^+^ MAIT Cells

**Figure 2:**
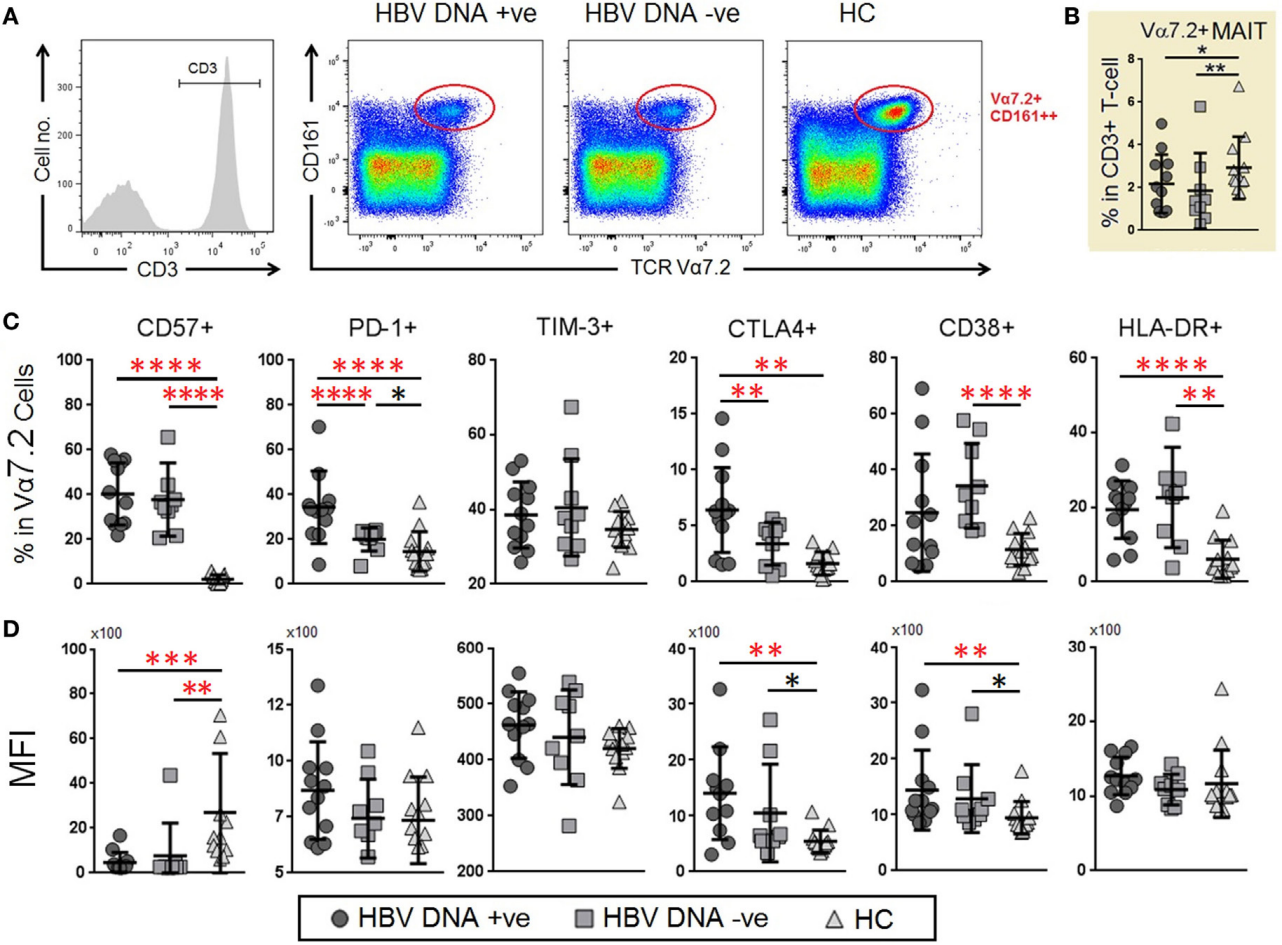
Frequencies and levels of immune senescence, exhaustion, and activation markers expressed on mucosal-associated invariant T (MAIT) cells between chronic hepatitis B virus (HBV)-infected patients with (G1, circle) and without (G2, square) HBV-DNAemia and healthy control (HC) (G3, triangle). **(A)** The gating strategy to identify CD3^+^TCR Vα7.2^+^ MAIT cells and Vα7.2^+^CD161^−^ T cells. **(B)** Comparison of T cell receptor (TCR) Vα7.2^+^ cells frequencies across the three groups comparison of percentage. **(C)** Expression levels [mean fluorescence intensity (MFI)] **(D)** of CD57, PD-1, TIM-3, CTLA-4 CD38 and HLA-DR on TCR Vα7.2^+^ MAIT cells between the three patient groups. Levels of surface markers were compared across the three patient groups and *post hoc* Mann–Whitney *U* tests were then performed for those biomarkers with a Kruskal–Wallis test *P* value of <0.05 (*P < 0.05, **<0.01, ***<0.001, and ****<0.0001). *P*-values remained significant after Benjamini–Hochberg correction of multiple comparisons (marked in red*).

To understand the response of MAIT cells and their potential role in chronic HBV infection, we examined the profile of co-expression of TCR iVα7.2 and CD161 on CD3^+^ lymphocytes (Figure 2A). Unlike CD4^+^ and CD8^+^ T cells (Figure S1 in Sup-plementary Material), the frequencies of TCR iVα7.2^+^ CD161^+^ subset were significantly reduced in the peripheral blood of chronic HBV-infected groups as compared to HCs (*P* < 0.05) (Figure 2B). In addition, there was an increase in the frequencies of CD57, PD-1 and CTLA-4 as well as HLA-DR expression in both HBV-DNA +ve and DNA −ve patient groups as compared to HCs. Of note, the HBV patients generally had a ~30-fold higher percentage of CD57^+^ MAIT cells as compared to HCs. Furthermore, the expression of PD-1 and CTLA-4 was significantly higher in HBV-DNA +ve patients as compared to HBV-DNA −ve patients (*P* < 0.00001 and *P* < 0.05, respectively). The percentage of PD-1 and CTLA-4 on MAIT cells were significantly higher in HBV-DNA +ve patients compared to HBV-DNA −ve patients (Figure 2C). In addition, the percentage and expression of CD38 and HLA-DR were generally higher among HBV patient groups than HCs, and there were an enhanced in the expression of CTLA-4 and CD38 (Figure 2D). We also found that the expression of PD-1 and TIM-3 were directly correlated in CD4^+^, CD8^+^ T cells, and MAIT cells (Figure S2 in Supplementary Material).

### Decrease of TCR iVα7.2^+^ MAIT Cells Was Associated with Chronic Immune Activation and Immunosenescence

**Figure 3:**
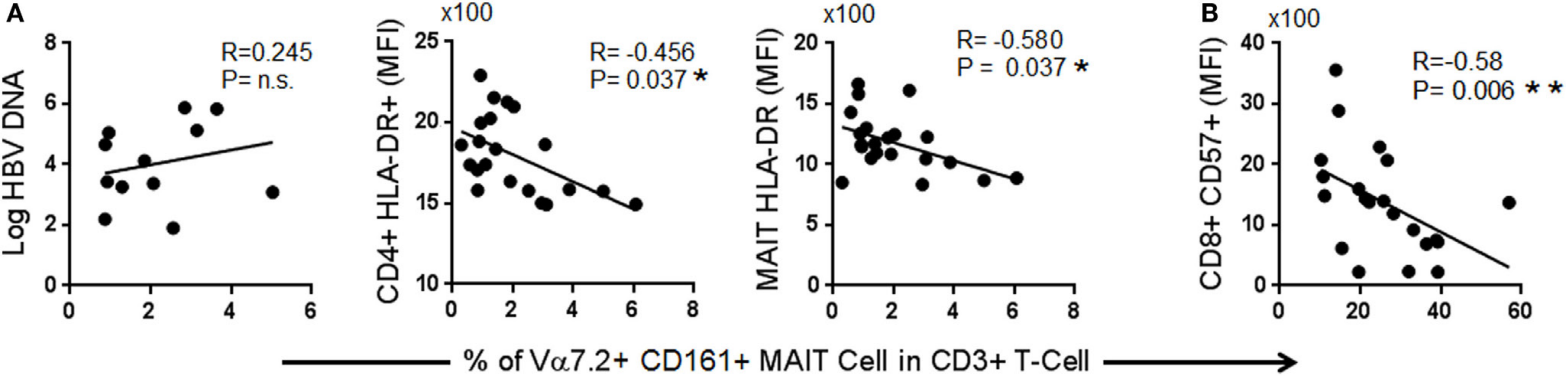
Spearman correlation between frequencies of T cell receptor Vα7.2^+^ CD161^+^ mucosal-associated invariant T (MAIT) with **(A)** markers of immune activation and **(B)** markers of immune senescence.

Given that the frequency of MAIT cells was decreased in chronic HBV infection, we next asked whether the degree of reduction was associated with the levels of viral replication or with markers of immunosenescence or chronic immune activation. We compared the frequencies of MAIT cells with levels of HBV-DNAemia, CD57, PD-1, CTLA-4, and HLA-DR using Spearman’s rank test. The frequency of MAIT cells did not correlate with the levels of HBV plasma DNAemia, but inversely correlated with expression (MFI) levels of HLA-DR on CD4^+^ T-cells and MAIT cells as well as with expression levels of CD57 on CD8^+^ T-cells (Figure 3). These data indicate that the depletion of MAIT cells may not directly due to HBV infection but rather due to persistent immune activation and immune senescence induced by chronic HBV infection.

### Expression of PD-1 and CTLA-4 on MAIT Cells Correlated Positively with the Plasma Levels of HBV-DNA

**Figure 4:**
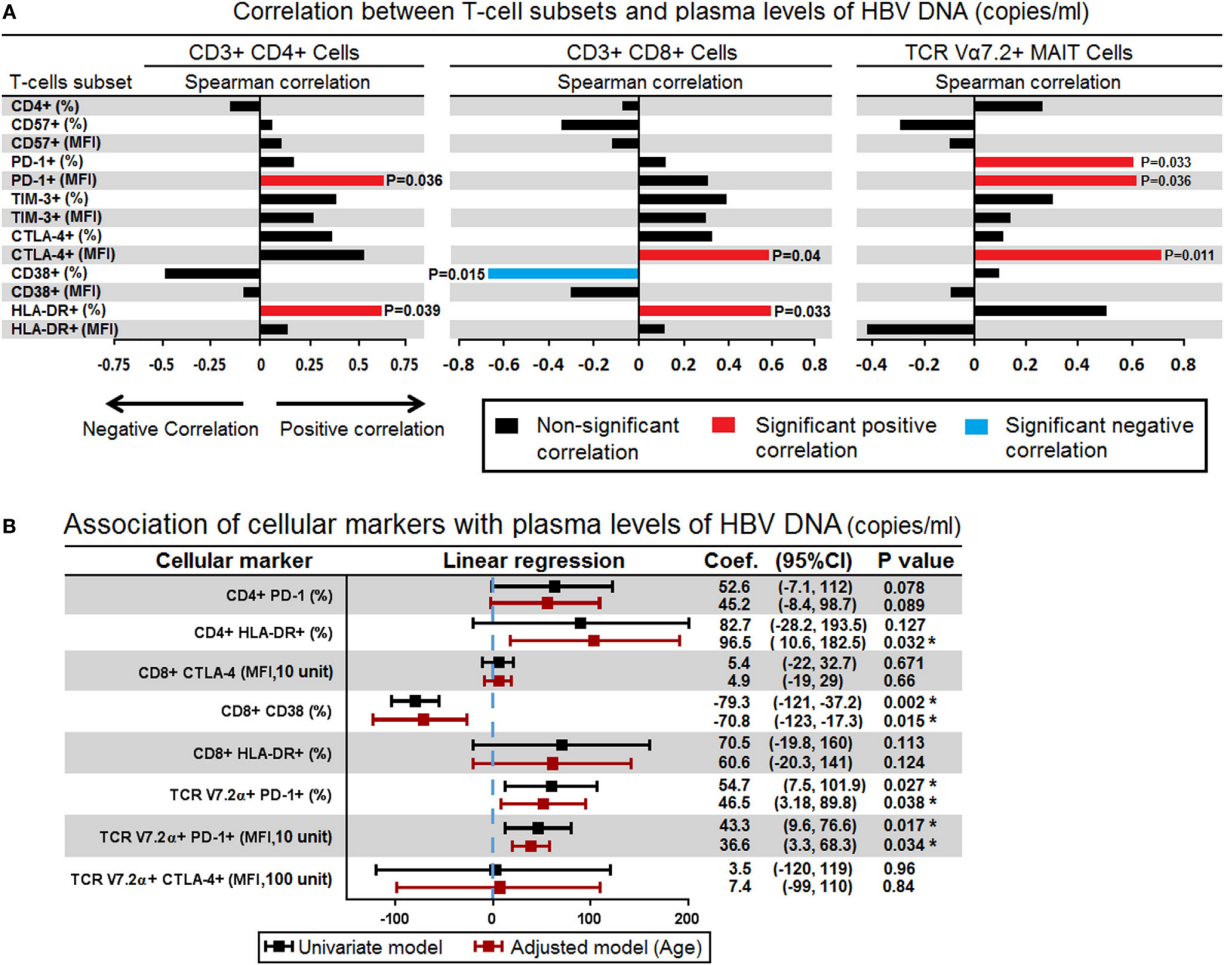
**(A)** Spearman correlations between surface markers in CD4^+^ (left panel), CD8^+^ (middle panel), and mucosal-associated invariant T (MAIT) cells (right panel) with level of plasma hepatitis B virus (HBV)-DNA. The bar represents the strength of association (*r* values) where red bar represents significant positive correlation, blue bar represent significant negative association and black bar represents *P* value > 0.05 (non-significant association) (<0.05, **<0.01, ***<0.001, and ****<0.0001). **(B)** Association of all surface markers that showed significant correlation with plasma HBV-DNA levels were assessed in simple logistic regression model and adjusted for age. Coefficient values below or above threshold levels were displayed in a forest plot; median and 95% CI were calculated. CI, confidence interval (*P < 0.05).

In the HBV-DNA +ve patients, we examined the immune correlate that could be associated with the plasma levels of HBV-DNAemia. In order to answer this question, using Spearman’s rank test, we compared the plasma levels of HBV-DNA (copies/μL) with percentages and expression (MFI) of CD57, PD-1, TIM-3, CTLA-4, CD38, and HLA-DR on CD4^+^, CD8^+^ T-cells and TCR Vα7.2^+^ CD161^+^ MAIT cells. Our analyses showed that the plasma levels of HBV-DNA positively correlated with the MFI of PD-1 (*r* = 0.61, *P* = 0.036) and frequency of HLA-DR expression on CD4^+^ T-cells (*r* = 0.00601, *P* = 0.039); MFI of CTLA-4 (*r* = 0.587, *P* = 0.045) and frequency of HLA-DR expression (*r* = 0.601, *P* = 0.039) on CD8^+^ T-cells and well as frequency of PD-1 expression (*r* = 0.601, *P* = 0.039), MFI of PD-1 (*r* = 0.608, *P* = 0.036), and MFI of CTLA-4 (*r* = 0.699, *P* = 0.011) on TCR Vα7.2^+^ CD161^+^ MAIT cells. Interestingly, we also found that the percentage of CD8^+^CD38^+^ T-cells inversely correlated with plasma levels of HBV-DNA (*r* = −0.678, *P* = 0.015) (Figure 4A).

Given that these surface markers may also be influenced by age, next we investigated the association between these surface markers and plasma levels of HBV-DNA using linear regression model and adjusted by age. We found that frequency and expression (MFI) of PD-1 on TCR Vα7.2^+^ CD161^+^ MAIT cells were associated with plasma levels of HBV-DNA; every 1% increase in the frequency of PD-1 expressing cells and 10 unit increase in the MFI of PD-1 were associated with increased plasma HBV-DNA levels by an average of 46.5 (*P* = 0.038) and 36.2 copies/μL (*P* = 0.034), respectively. In contrast every 1% increase in the frequency of CD8^+^CD38^+^ T-cells was associated with decreased plasma HBV-DNA levels by an average of 70.8 copies/μL (*P* = 0.015) (Figure 4B). These results indicated that the expression PD-1 on TCR iVα7.2 MAIT cells and CD38 on CD8 cells played significant role in controlling the chronic HBV infection.

### Expression of PD-1 on TCR Vα7.2 MAIT Cells Correlated Inversely with Levels of Plasma ALT

**Figure 5:**
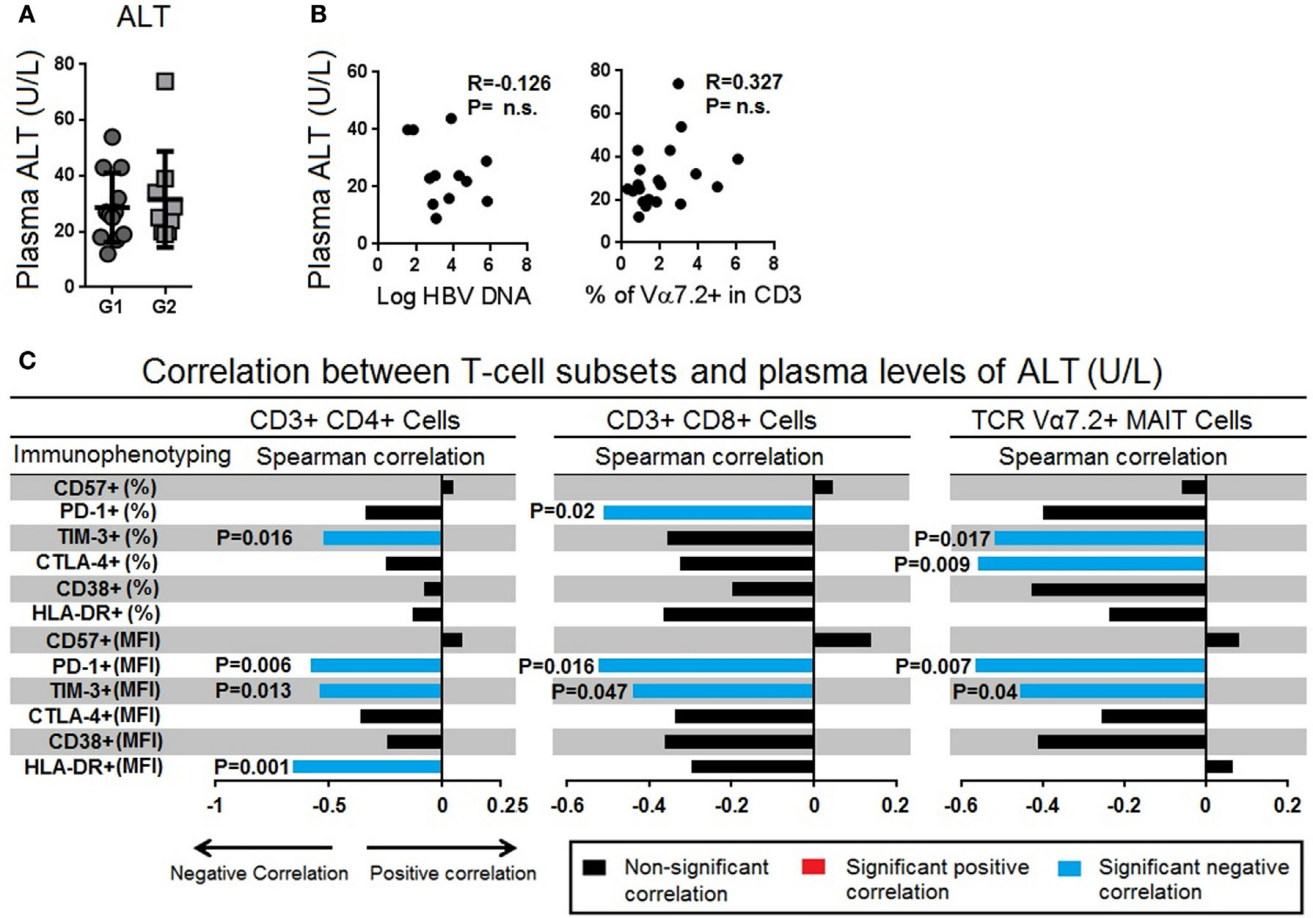
**(A)** Comparison of serum alanine transaminase (ALT) levels between chronic hepatitis B virus (HBV)-infected patients with HBV-DNAemia (circle) and without HBV-DNAemia (square). **(B)** Spearman correlation between serum ALT levels and frequencies of T cell receptor (TCR) Vα7.2^+^ CD161^+^ mucosal-associated invariant T (MAIT) cells. **(C)** Spearman correlation between frequencies and expression levels of CD57, PD-1, TIM-3, CTLA-4 CD38, and HLA-DR with serum level of ALT. The bar represents the strength of association (*r* values) where red bar represents significant positive correlation, blue bar represent significant negative association and black bar represents *P* value > 0.05 (non-significant association) (<0.05, **<0.01, ***<0.001, and ****<0.0001).

It has previously been shown that the levels of serum alanine aminotransferase (ALT) are linked to a more robust immune response that can lead to seroclearance of HBeAg as well as decreased plasma HBV-DNA loads (32, 33). Here, we failed to find a correlation between ALT levels and plasma levels of HBV-DNA (Figure 5A). The ALT levels were also not correlated with frequencies of TCR Vα7.2^+^ MAIT cells (Figure 5B). However, the frequencies and MFI of several inhibitory molecules, especially TIM-3 and PD-1 expressed on CD4^+^, CD8^+^ T cells, and TCR Vα7.2^+^ MAIT cells, were found to inversely correlate with the levels of ALT. These results further supported our finding that PD-1 plays an important role in persistent HBV infection (Figure 5C).

### TCR iVα7.2^+^ MAIT Cells of Chronic HBV-Infected Patients Were Functionally Impaired in Granzyme-B and IFN-γ Production

**Figure 6:**
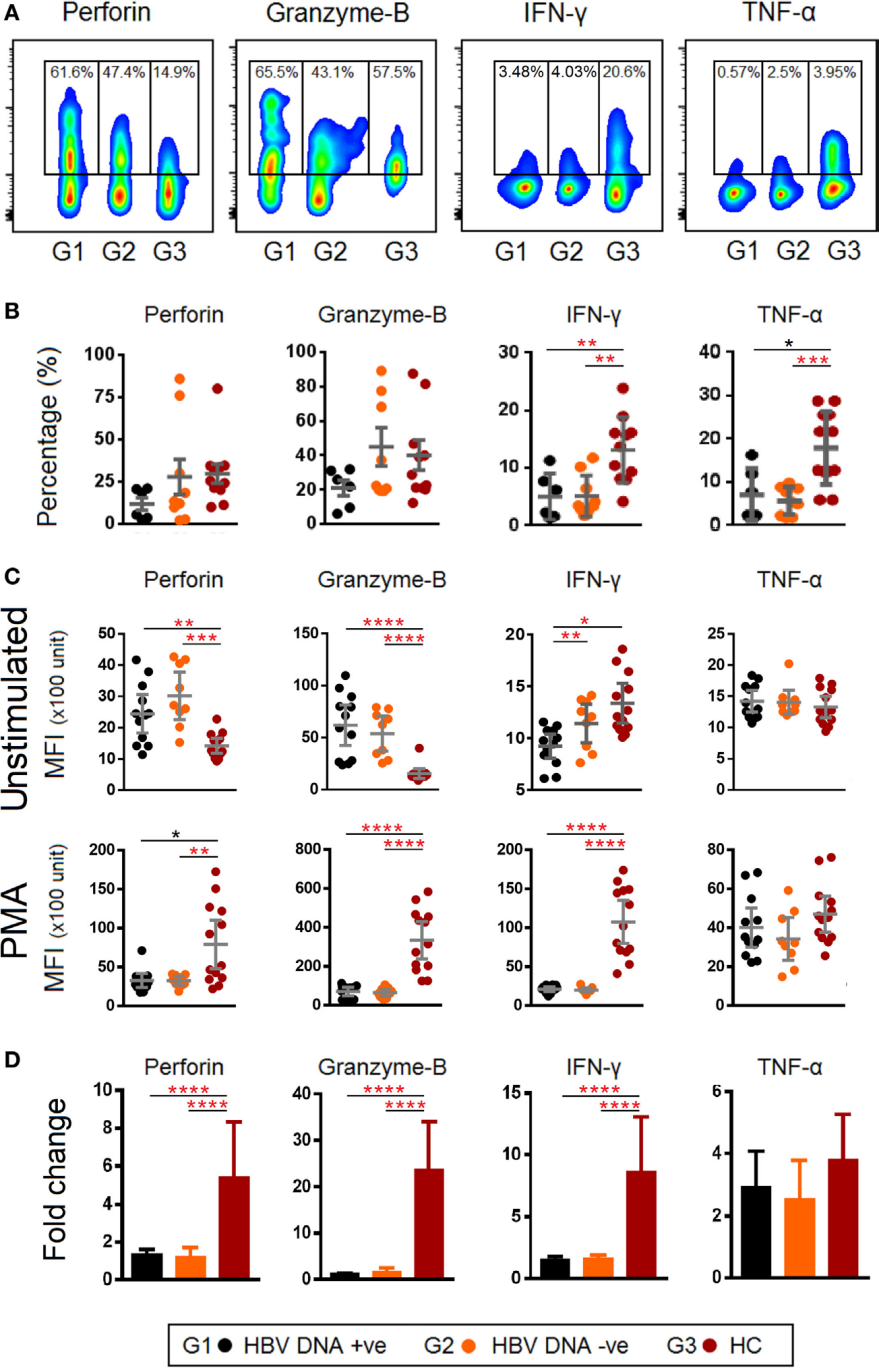
**(A)** Representative density dot plots for perforin, granzyme-B, IFN-γ, and TNF-α (without stimulation) in T cell receptor (TCR) Vα7.2^+^ mucosal-associated invariant T (MAIT) cells in chronic hepatitis B virus (HBV)-infected patients with HBV-DNAemia (G1) and without HBV-DNAemia (G2) and healthy control (HC) (G3). **(B)** Comparisons of percentages of perforin, granzyme-B, IFN-γ, and TNF-α in TCR Vα7.2^+^ across the three groups. **(C)** Comparisons of expression levels [mean fluorescence intensity (MFI)] of perforin, granzyme-B, IFN-γ, and TNF-α in TCR Vα7.2^+^ before and after PMA stimulation. **(D)** Expression (measured as fold change of MFI) of perforin, granzyme-B, IFN-γ, and TNF-α in TCR Vα7.2^+^ after PMA stimulation. Levels of cytokine expression were compared across the three patient groups and *post hoc* Mann–Whitney *U* tests were subsequently performed for those biomarkers using a Kruskal–Wallis test (level of significance *P* < 0.05) (*P < 0.05, **<0.01, ***<0.001, and ****<0.0001). *P*-values remained significant after Benjamini–Hochberg correction of multiple comparisons (marked in red*).

Given that the frequency of TCR Vα7.2^+^ MAIT cells was reduced and expressed higher levels of PD-1 and CTLA-4 in chronic HBV-infected patients, we examined if this phenotype was associated with functional impairment by performing intracellular staining for perforin, granzyme-B, IFN-γ, and TNF-α (Figure 6A). Our results showed that the frequencies of cells producing IFN-γ, TNF-α were lower (Figure 6B), while expression (MFI) of perforin and granzyme-B were higher in chronic HBV-infected patients as compared to HCs (Figure 6C). Furthermore, results from *ex vivo* PMA stimulation experiments showed that the TCR Vα7.2^+^ MAIT cells of chronic HBV-infected patients had lower expressions of perforin, granzyme-B, and IFN-γ compared to HCs (Figure 6C). Analyses using Wilcoxon signed rank test for the samples before and after PMA stimulation showed that the production of cytokines were generally increased in all patients groups after PMA stimulation except for granzyme-B in HBV-infected patients where changes were not significantly altered after PMA stimulation (Figure S3 in Supplementary Material). Further analyses also showed that levels of perforin, granzyme-B and IFN-γ were ~4-, ~25-, and ~5-fold lower, respectively, in HBV-infected patients compared to HCs (Figure 6D). Together, these results indicate that there was a functional impairment in TCR Vα7.2^+^ MAIT cells.

## Discussion

Upregulation of co-inhibitory molecules on CD8^+^ and CD4^+^ T-cells has been shown to be associated with persistent viral replication in the setting of chronic viral infections (34–39), however the role of immune exhaustion in MAIT cells remain less well understood. Given that the TCR Vα7.2^+^ MAIT cells are found in circulation and enriched in mucosal compartment and liver, we postulate that the levels of immune exhaustion on MAIT cells are secondary to ongoing HBV infection and may hamper HBV viral clearance. In our study, we found that the frequencies of MAIT cells were reduced among chronic HBV-infected patients. More importantly, the immune checkpoint PD-1 was over expressed in chronic HBV-infected patients with HBV-DNAemia compared to other groups and the frequency and expression levels of PD-1 on MAIT cells were predictive of the levels of HBV-DNA in plasma. MAIT cells from chronic HBV-infected patients were producing low levels of granzyme-B and IFN-γ after stimulation.

Mucosal-associated invariant T cells are activated either by recognizing riboflavin metabolites produced by a wide range of microbes (including bacteria and fungi) presented on MHC-Ib-related protein (MR1) (40, 41), or independently of the TCR by stimulation by pro-inflammatory cytokine such as IL-12 and IL-18 (42, 43), which could occur during viral infections (23, 27). Indeed, TLR8 agonists have been shown to activate intrahepatic MAIT cells by inducing the expression of IL-12 and IL-18 by intrahepatic monocytes (42). Upon activation, MAIT cells respond rapidly with cellular cytotoxicity (44) as well as secretion of pro-inflammatory and antiviral cytokines such as IFN-γ, and TNF-α (19, 45). Given the important role of MAIT cell responses in the mucosa, we also found a decreased frequency of MAIT cells in the blood of patients chronically infected with HBV.

The frequency of MAIT cells was not correlated with the levels of HBV plasma DNAemia, but correlated with expression of immune activation and immune senescence markers on CD4^+^ and CD8^+^ T cells, suggesting the depletion of MAIT cell could likely be due to immune activation and senescence driven by chronic HBV infection. PD-1 was upregulated on MAIT cells, suggesting that they may be functionally exhausted in chronic HBV infection. This was also evident from intracellular staining experiments that indicated a marked reduction in the induction of granzyme-B and IFN-γ among HBV-infected patients. Furthermore, the MFI of IFN-γ expressing MAIT cells in HBV-DNA −ve patients were higher compared to HBV-DNA +ve patients indicating that the MAIT cells’ functions may be partially normalized in HBV-DNA −ve patients. These findings highlight the importance of IFN-γ in seroclearance of HBV-DNA.

PD-1 on the other hand, is a negative regulator of immune responses and is predominantly expressed on activated T cells (46, 47), while the ligand of PD-1, PD-1L is expressed on activated antigen presenting cells such as DCs (46, 47). The interaction between PD-1 and PD-L1 transmits a bidirectional inhibitory signal that limits downstream activation, proliferation, and cytokine production (48, 49). Though expression of PD-1 has been associated with tissue protection against severe injury by exacerbated immune responses (48, 49), the same mechanism has also been implicated in facilitating residual viral replication and persistent viral infections as the blockade of PD-1 restores T-cells functions against chronic viral infections including lymphocytic choriomeningitis virus (50), Epstein–Barr virus (51), and HIV, where the viral load is reduced in infected humanized mice (52). In this study, despite the elevated expression of several markers of T-cell dysfunction on CD4^+^ and CD8^+^ T cells and MAIT cells in patients with chronic HBV infection, the frequency of PD-1 expressing MAIT cells was the only marker that clearly differentiated between HBV-infected patients with HBV-DNAemia, those without HBV-DNAemia, and HC. Furthermore, there was a positive correlation of both frequency and level of expression of PD-1 on MAIT cells with plasma levels of HBV-DNA and a negative correlation with serum ALT. Taken together, our results suggest that MAIT cells may play a role in controlling HBV replication, and that elevated HBV-DNA levels results in MAIT cell depletion and exhaustion; while suppression of HBV replication in the liver improves MAIT cell function the numbers do not appear to recover in the absence of DNAemia.

Plasma HBV-DNA levels are an important predictor for HBV disease progression to clinical end-point. Both case–control and cohort studies have shown a significant, level-dependent association between HBV-DNA and the subsequent risk of cirrhosis (53–55) and HCC (56–58). Intervention studies also showed the same association between levels of serum HBV-DNA and disease progression seen on liver biopsy (59, 60). Our results showed that the level of PD-1 expression on MAIT cells was predictive of the plasma HBV-DNA levels, which suggests that MAIT cell functions may be restored by blocking PD-1 expression. It remains to be seen whether such a therapy would have any effect on the risk of HBV clinical end-point disease. Further studies by blocking co-inhibitory molecule expression in HBV-infected patients may be warranted.

In future studies, it will be important to study MAIT cells in the hepatic compartment. While peripheral blood MAIT cells were found to be depleted in this study, the intrahepatic population appears to be preserved in chronic hepatitis B infection, although not in end-stage disease (37). It remains to be determined whether intrahepatic MAIT cells in chronic hepatitis B infection are functionally exhausted, as they are in the periphery, and whether they can still respond normally to TCR and cytokine-mediated stimulation.

In summary, we have characterized for the first time the effect of chronic HBV infection on peripheral blood MAIT cells by investigating the expression of co-inhibitory molecules and cytotoxicity molecules, and level of cytokines production. These findings suggest a possible role for MAIT cells in the control of HBV replication, and that failure to control infection results in MAIT cell functional exhaustion.

## Ethics Statement

This study was carried out in accordance with the recommendations of “name of guidelines, name of committee” with written informed consent from all subjects. All subjects gave written informed consent in accordance with the Declaration of Helsinki. The protocol was approved by the Medical Ethics Committee of the UMMC Kuala Lumpur, Malaysia (MEC201311-0496).

## Author Contributions

YY, AS, HT, RB, PE, and ES conceived and designed the experiments; TY, AS, RB, and PE carried out experiments and data analysis; YY, ES, MR, JR, and ML documented the findings; AK, MR, JU, and VaV selected patient samples and collected the clinical data for patients. RV, IC, AM, JU, VVe, JR, ML, and ES wrote the manuscript; VVe, AA, AM, JU, and ML contributed reagents and analysis tools; AA, RV, JU, IC, VVe, VaV, and ML provided critical inputs to the manuscript.

## Conflict of Interest Statement

The authors declare that the research was conducted in the absence of any commercial or financial relationships that could be construed as a potential conflict of interest.

## Supplementary Material

Figure S1 Comparison of percentages of CD4^+^ and CD8^+^ T-cells between chronic hepatitis B virus (HBV)-infected patients with (circle) and without (square) HBV-DNAemia and healthy control (triangle).

Figure S2 Spearman correlation between percentage and MFI of TIM-3 and PD-1 in CD4^+^, CD8^+^ and MAIT cells.

Figure S3 Changes in perforin, granzyme-B, IFN-γ and TNF-α expression following stimulation by PMA. Data were analysed using Wilcoxon matched-pairs test for paired analyses within each study group. **P* < 0.05, ***P* < 0.01, ****P* < 0.001.
