# Supplementary figures and images for: Hyper-Expression of PD-1 Is Associated with the Levels of Exhausted and Dysfunctional Phenotypes of Circulating CD161++TCR iVα7.2+ Mucosal-Associated Invariant T Cells in Chronic Hepatitis B Virus Infection
Source: Front Immunol. 2018 Mar 19;9:472. doi: 10.3389/fimmu.2018.00472 (PMC5868455; doi:10.3389/fimmu.2018.00472)

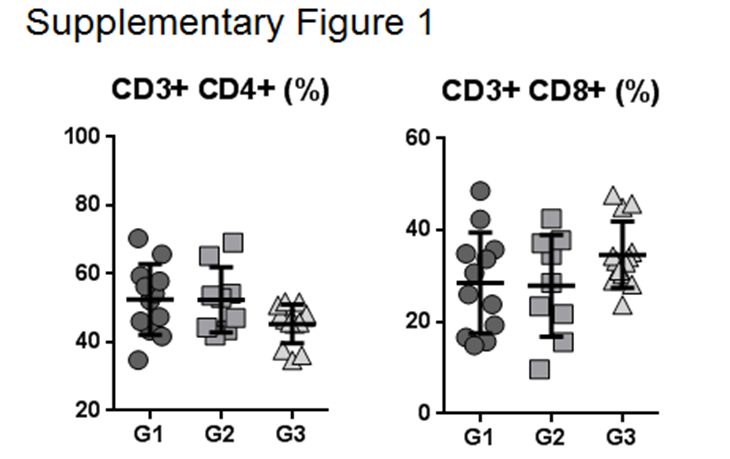

Supplement: Figure S1 — Comparison of percentages of CD4+ and CD8+ T-cells between chronic hepatitis B virus (HBV)-infected patients with (circle) and without (square) HBV-DNAemia and healthy control (triangle). [file Image_1.tif]

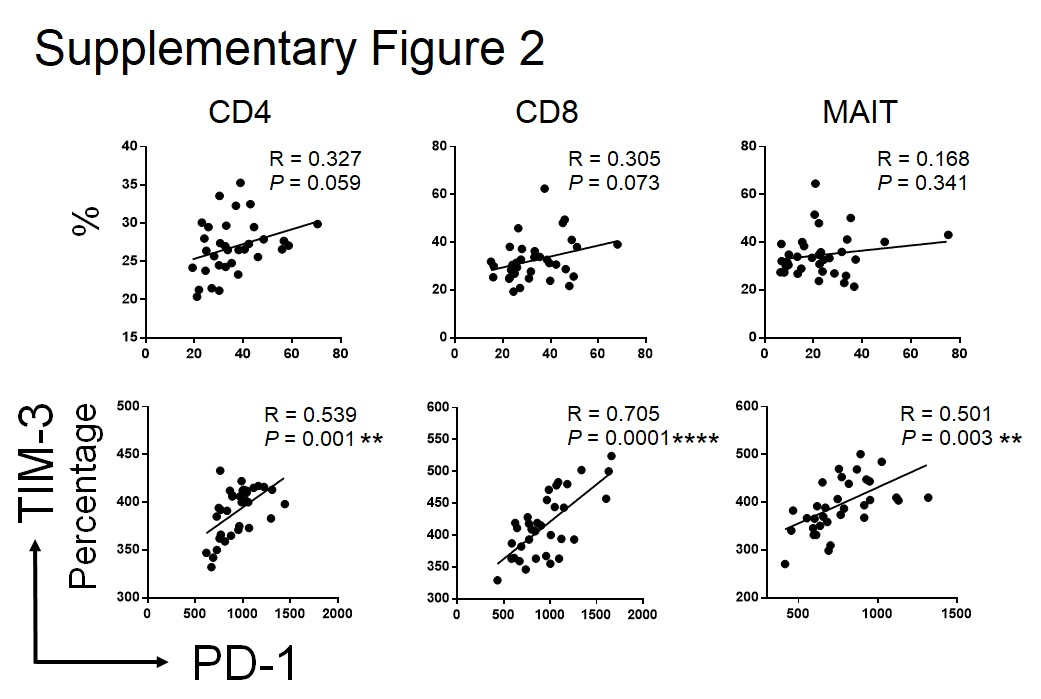

Supplement: Figure S2 — Spearman correlation between percentage and MFI of TIM-3 and PD-1 in CD4+, CD8+ and MAIT cells. [file Image_2.tif]

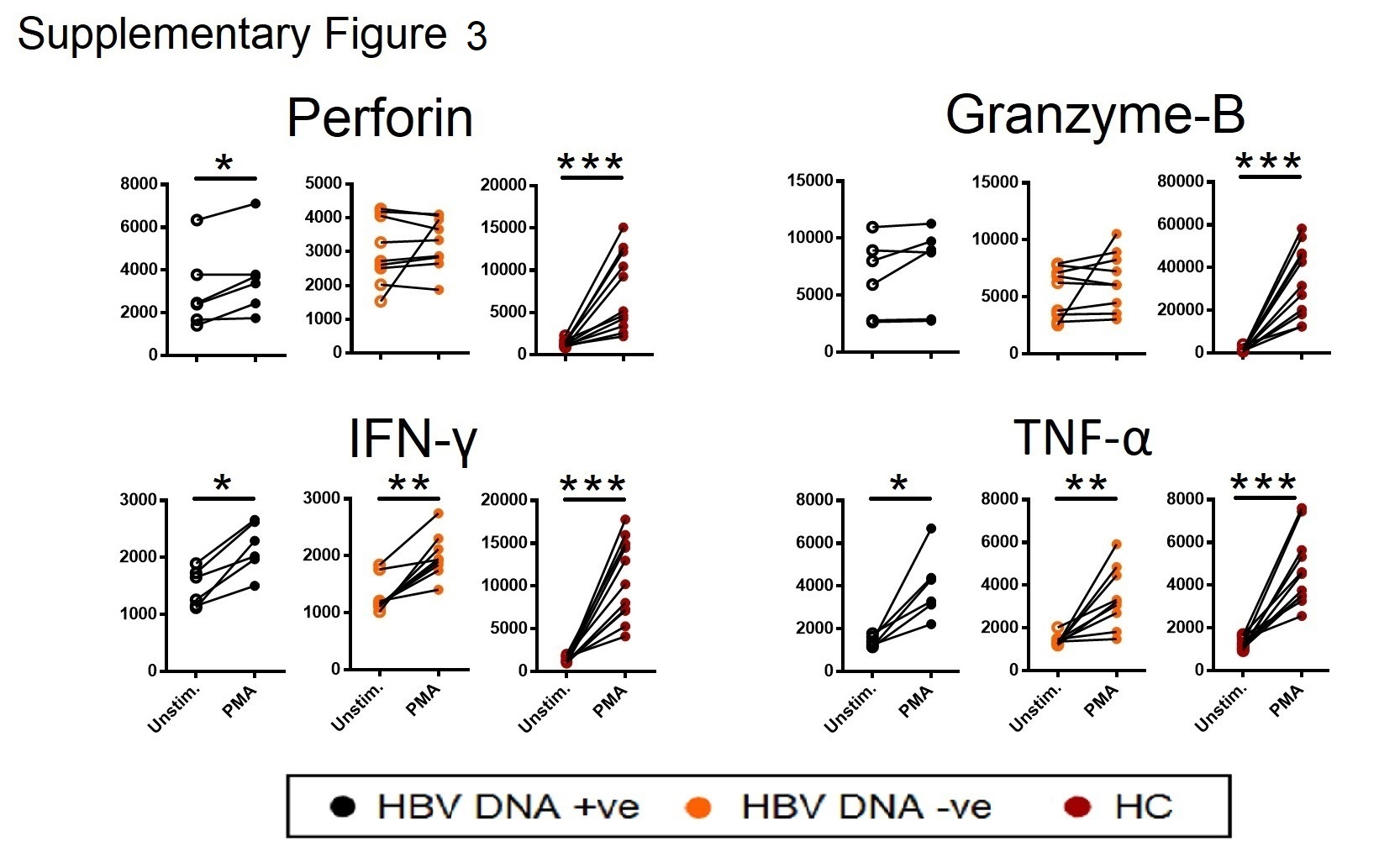

Supplement: Figure S3 — Changes in perforin, granzyme-B, IFN-γ and TNF-α expression following stimulation by PMA. Data were analysed using Wilcoxon matched-pairs test for paired analyses within each study group. *P < 0.05, **P < 0.01, ***P < 0.001. [file Image_3.tif]
